# Melorheostosis Involving the Carpal and Metacarpal Bones: Diagnostic Insights From Multimodal Imaging

**DOI:** 10.1002/ccr3.73526

**Published:** 2026-10-04

**Authors:** Zhiwei Zhang, Zhi Deng, Wen Yang, Aiguo Zou, Pengyuan Leng, Yuewen Peng

**Affiliations:** ^1^ Orthopedics Department Yueyang People's Hospital Yueyang City Hunan Province China; ^2^ Traditional Chinese Medicine Department Yueyang People's Hospital Yueyang City Hunan Province China

**Keywords:** case report, hand, magnetic resonance imaging, MAP2K1, melorheostosis, wrist

## Abstract

Multimodal imaging enables non‐invasive diagnosis of carpal melorheostosis, avoiding biopsy. Two‐year follow‐up confirms stability.

## 1 Introduction

Melorheostosis, also known as Léri‐Joanny syndrome, is a rare, non‐hereditary, congenital sclerotic bone dysplasia with an estimated prevalence of approximately 0.9 per million individuals [1]. First described by Léri and Joanny in 1922 [2], it is classically known for a distinctive radiological pattern resembling “melting wax dripping down” the bone surface [3]. The pathophysiology has been recently elucidated through the identification of somatic activating mutations in MAP2K1 (encoding MEK1), which lead to constitutive activation of the MAPK/ERK pathway and aberrant osteoblast activity [4].

Although the lower limbs are predominantly affected, involvement of the upper extremities—particularly the small bones of the hand and wrist—is reported in < 10% of cases and often presents diagnostic ambiguity [5]. Freyschmidt's review of 23 cases identified only five cases involving the upper extremity, with radiological patterns classified into five subtypes: classic candle‐wax, osteoma‐like, osteopathia striata‐like, myositis ossificans‐like, and mixed [1]. Additionally, Yu et al. classified melorheostosis into endosteal, periosteal, parosteal, and mixed types based on the direction of lesion spread [6].

The diagnosis of melorheostosis relies primarily on radiological findings. However, asymptomatic or incidental cases involving the wrist require careful differentiation from malignant or infectious processes to avoid unnecessary invasive procedures. Recent case reporting—such as the pediatric multifocal case by Na et al. [7]—has enhanced understanding of diagnosis and management, particularly in upper limb presentations.

Herein, we present a case of asymptomatic melorheostosis involving the second metacarpal, lunate, and triquetrum in an adult, discovered incidentally after trauma. We emphasize the role of multimodal imaging in securing a non‐invasive diagnosis, classify the case within the Freyschmidt and Yu et al. frameworks, and integrate recent molecular insights to provide a comprehensive clinical perspective.

## 2 Case Presentation

### 2.1 Clinical Timeline

Day 0 (Injury): A 21‐year‐old right‐hand‐dominant male sustained a fall onto his outstretched left hand during recreational sports. He reported mild wrist discomfort but no prior pain or functional limitation.

Day 1 (Initial Visit): Presented to the orthopedics clinic. Physical examination revealed localized tenderness over the left thenar eminence. No erythema, deformity, or sensory deficits were noted. Range of motion was full and painless.

Day 1 (Imaging): Initial radiographs were performed to exclude fracture, revealing incidental sclerotic lesions. Subsequent CT and MRI were conducted for further characterization.

Month 24 (Follow‐up): The patient remained asymptomatic with no functional impairment. No repeat imaging was performed as the absence of symptoms did not warrant radiation exposure.

### 2.2 Diagnostic Assessment

#### 2.2.1 Physical Examination

No deformity was observed in the left hand or wrist. Physical examination revealed tenderness over the thenar eminence without erythema or deformity. Neurovascular assessment showed intact sensation and capillary refill. The range of motion of the wrist and finger joints was normal. Neurovascular status was intact.

### 2.3 Laboratory Findings

Routine blood work was unremarkable. Complete blood count, C‐reactive protein (CRP: 2 mg/L, reference 0–10 mg/L), and erythrocyte sedimentation rate (ESR: 3 mm/h, reference 0–15 mm/h) were within normal limits. Serum alkaline phosphatase (ALP) was 51 U/L (reference: 35–105 U/L), and calcium was 9.0 mg/dL (reference: 8.4–10.2 mg/dL). These findings supported a non‐inflammatory, non‐metabolic bone process.

#### 2.3.1 Imaging Findings

##### 2.3.1.1 Radiographs

Posteroanterior views of the left hand demonstrated irregular, linear hyperdense sclerosis involving the second metacarpal, lunate, triquetrum, and distal ends of the radius and ulna. The lesions exhibited a dense, “ivory‐like” quality with a characteristic flowing pattern along the bone contours, suggestive of “dripping candle wax.” No periosteal reaction or soft tissue swelling was observed (Figure 1).

**FIGURE 1:**
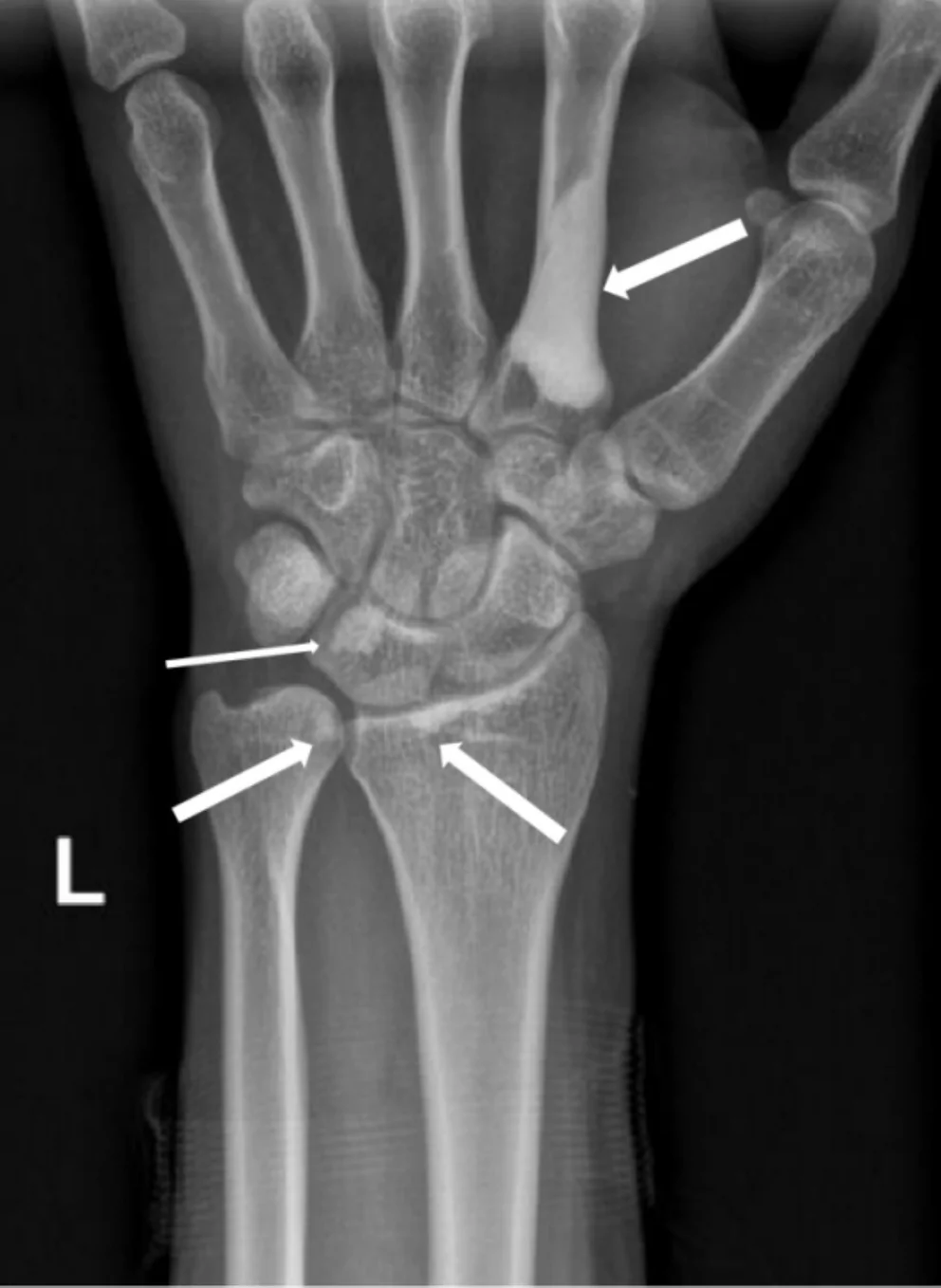
X‐ray features of melorheostosis in the left hand (Posteroanterior view). The radiograph shows flowing, hyperdense sclerotic lesions (arrows) in the second metacarpal, lunate, triquetrum, and distal ulna and radius.

##### 2.3.1.2 Computed Tomography (CT)

Axial and coronal reformats provided exquisite osseous detail, confirming irregular endosteal and intracortical hyperdensity. Cortical thickening and partial medullary cavity obliteration were evident in the affected bones. The sclerotic areas blended seamlessly with the cortex, creating a “melted wax” appearance. Adjacent joints were preserved (Figure 2).

**FIGURE 2:**
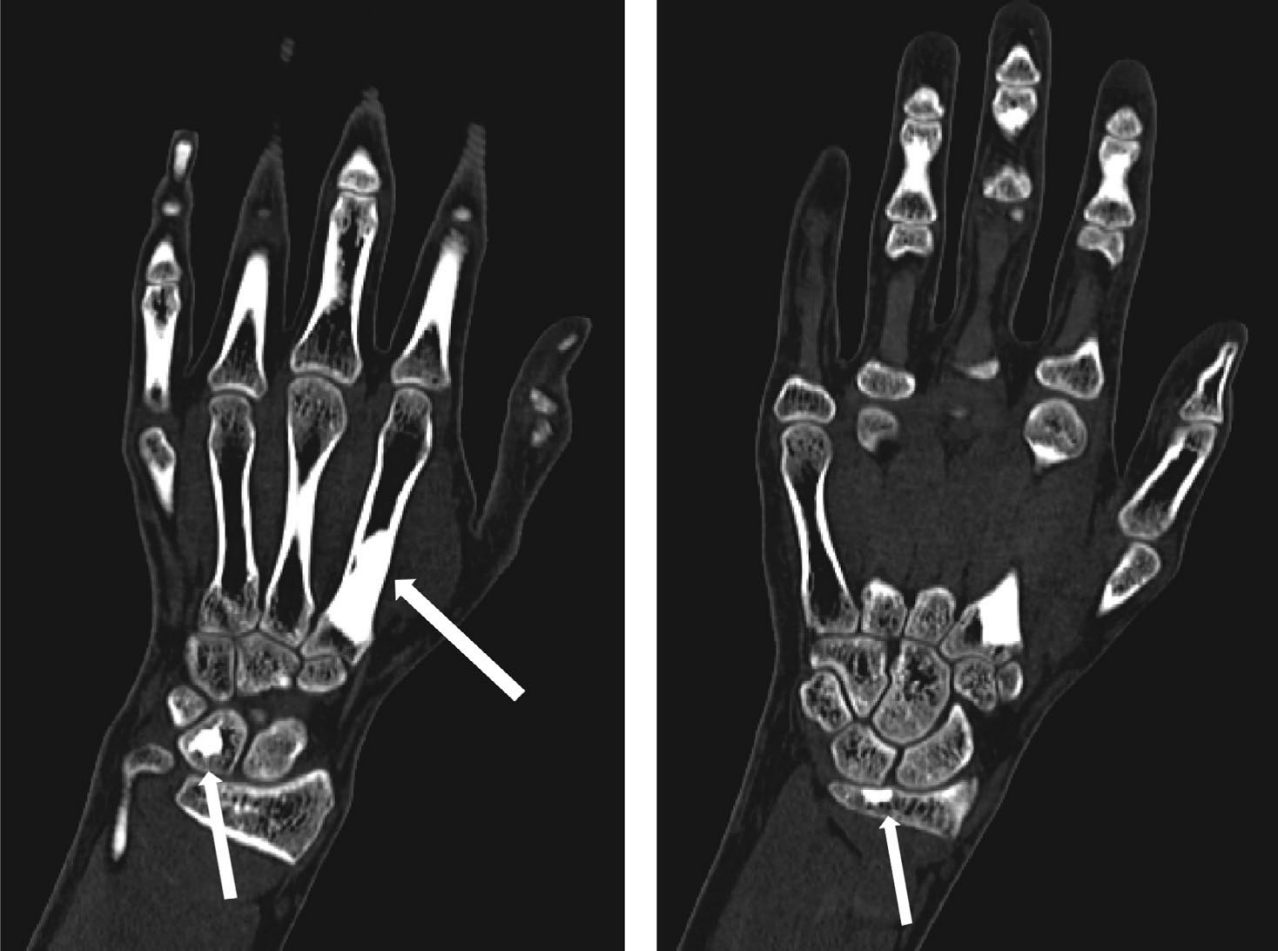
CT features of melorheostosis in the left hand (Coronal reformats). Coronal CT images demonstrate the endosteal and intracortical location of the dense sclerosis (arrows), with cortical thickening and medullary encroachment.

##### 2.3.1.3 Magnetic Resonance Imaging (MRI)

Coronal T1‐weighted (T1WI) and fat‐suppressed T2‐weighted (T2WI) sequences revealed homogeneous, markedly low signal intensity within the sclerotic lesions, isointense to cortical bone. Crucially, there was no associated bone marrow edema, soft tissue mass, or joint effusion. The surrounding bone marrow signal was normal (Figure 3).

**FIGURE 3:**
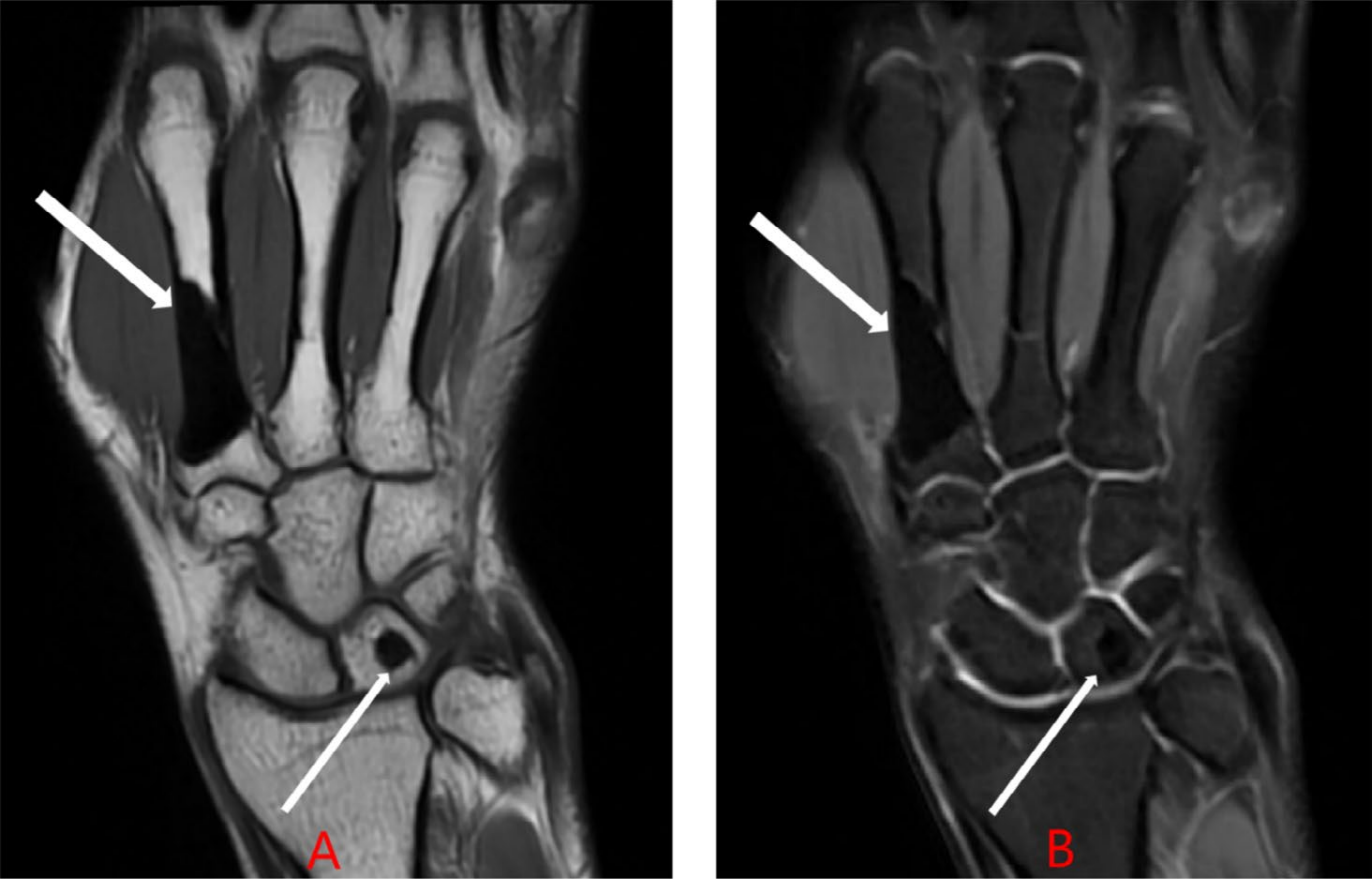
MRI characteristics of the lesions (Coronal T1WI and fat‐suppressed T2WI). (A) Coronal T1‐weighted image shows the lesions (arrows) as areas of homogeneous low signal intensity. (B) Coronal fat‐suppressed T2‐weighted image confirms the persistently low signal intensity (arrows) within the sclerotic areas. Note the absence of marrow edema or soft tissue abnormality.

### 2.4 Diagnosis

Based on the classic radiographic “wax‐flow” pattern, CT confirmation of cortical sclerosis, and the absence of aggressive features on MRI (specifically, no marrow edema or mass), a definitive diagnosis of melorheostosis was established. The lack of systemic symptoms and normal laboratory values further supported a benign, quiescent process. According to Freyschmidt's radiological classification [1], this case is most consistent with the classic candle‐wax subtype; per Yu et al.'s classification [6], it represents the endosteal type.

### 2.5 Therapeutic Intervention

The patient was reassured about the benign, non‐neoplastic nature of the condition. Given the asymptomatic nature, conservative management was chosen, involving patient education and periodic monitoring. No pharmacological or surgical intervention was indicated. He was advised to undergo periodic clinical follow‐up and to seek consultation if pain or functional limitation develops.

### 2.6 Follow‐Up and Outcomes

At the 2‐year follow‐up, the patient remained completely asymptomatic. No repeat imaging was performed during this period, as the absence of clinical symptoms did not warrant radiation exposure. Limitation: The lack of radiographic confirmation of stability is a limitation; however, the sustained asymptomatic clinical course over 2 years strongly supports a benign, quiescent process.

## 3 Discussion

### 3.1 Comparison With Previously Published Hand/Wrist Cases

This case contributes to the literature by detailing the multimodal imaging features of melorheostosis involving the carpal and metacarpal bones—a rare anatomical distribution. While Freyschmidt noted that only 5 of 23 cases (≈22%) involved the upper extremity [1], specific reports of lunate and triquetrum involvement are exceedingly scarce. Our findings align with Koca et al. [8], who emphasized that melorheostosis may present with heterogeneous radiological patterns, including an “osteoma‐like” form that can mimic parosteal osteosarcoma.

A particularly relevant recent case is that of Na et al. [7], who reported a 9‐year‐old male with multifocal melorheostosis of the right upper limb, involving the scapular coracoid process, humeral head, distal humerus, ulnar head, wrist, and metacarpals. That pediatric case exhibited a pathognomonic “melting wax drip” pattern and was classified as the endosteal subtype per Yu et al.'s classification [6]. Histopathological examination revealed irregular endosteal bone proliferation with densely arranged lamellar bone and enlarged/distorted Haversian canals. Notably, the pediatric patient underwent biopsy for definitive diagnosis, whereas our adult patient avoided invasive biopsy through the integration of multimodal imaging. Both cases remained asymptomatic over their respective follow‐up periods (6 months for Na et al.'s case [7]; 2 years for ours, albeit without repeat imaging in our case), reinforcing the management principle that asymptomatic melorheostosis requires monitoring only.

The comparison highlights two key distinctions: (1) age distribution—pediatric versus adult presentation; (2) lesion distribution—multifocal unilateral upper limb versus localized carpometacarpal involvement. These differences underscore the phenotypic spectrum of melorheostosis and validate the utility of non‐invasive imaging‐based diagnosis in cooperative adult patients.

### 3.2 Role of MRI in Avoiding Misdiagnosis

A critical teaching point from this case is the role of MRI in avoiding misdiagnosis. While the absence of marrow edema alone cannot entirely exclude a low‐grade malignancy [9], the combination of homogeneous low signal intensity on all sequences (reflecting dense sclerosis), the lack of a soft tissue mass, and the absence of periosteal reaction strongly favors a benign, inactive process. In contrast, osteosarcoma typically exhibits heterogeneous signal intensity, a soft tissue mass, and aggressive periosteal reaction [10]. Thus, the diagnosis here was secured by the overall clinical and radiological constellation rather than a single imaging feature.

### 3.3 Molecular Insights and Pathophysiology

Recent advances have transformed our understanding of melorheostosis from a purely radiological entity to a genetically defined disorder. Somatic activating mutations in MAP2K1 are identified in a significant subset of patients, leading to constitutive activation of the MEK–ERK pathway, which drives aberrant osteoblast proliferation and impaired mineralization [4]. While genetic testing was not performed in this asymptomatic patient—consistent with current clinical practice where diagnosis is imaging‐based—recognizing these molecular underpinnings is crucial. It explains the localized nature of the disease due to post‐zygotic mosaicism and opens avenues for potential targeted therapies in symptomatic cases [8].

### 3.4 Differential Diagnosis and Management

The differential diagnosis included osteosarcoma, chronic osteomyelitis, and osteopoikilosis. Osteopoikilosis, often associated with LEMD3 mutations, presents with multiple small, round sclerotic foci but lacks the linear, flowing morphology [11]. The management of melorheostosis is typically conservative for asymptomatic patients. Intervention is reserved for complications such as nerve entrapment, progressive deformity, or intractable pain [12]. Our case reinforces that conservative management with periodic surveillance is appropriate for incidental, asymptomatic lesions.

## 4 Conclusion

Melorheostosis involving the carpal and metacarpal bones can be diagnosed non‐invasively through integrated clinical and multimodal imaging findings. In this case, the classic radiographic “candle‐wax” pattern (Freyschmidt classic subtype [1]; Yu endosteal type [6]) and absence of aggressive MRI features, together with a 2‐year asymptomatic clinical course, confirmed a benign quiescent lesion and avoided biopsy. This case aligns with management principles for non‐progressive melorheostosis—monitoring only for asymptomatic patients [7, 12]—and underscores the need for sustained surveillance to better understand its natural history given phenotypic heterogeneity and theoretical long‐term risks [8].

## Author Contributions

**Zhiwei Zhang:** writing – original draft, writing – review and editing. **Zhi Deng:** investigation, resources. **Wen Yang:** project administration, investigation. **Aiguo Zou:** data curation, software. **Pengyuan Leng:** methodology. **Yuewen Peng:** project administration, supervision.

## Funding

The authors have nothing to report.

## Disclosure

Patient Perspective: The patient expressed satisfaction with the non‐interventional approach and reported no concerns during follow‐up.

## Ethics Statement

This case report was conducted in accordance with the Declaration of Helsinki. The publication of this case was approved by the Ethics Committee of Yueyang People's Hospital.

## Consent

Written informed consent was obtained from the patient for the publication of his clinical details and imaging studies.

## Conflicts of Interest

The authors declare no conflicts of interest.

## Data Availability

The data that support the findings of this study are available from the corresponding author upon reasonable request.
